# Mentalizing and emotion dysregulation in emerging adults during the COVID-19 pandemic: a pilot short-term longitudinal study

**DOI:** 10.1192/j.eurpsy.2022.953

**Published:** 2022-09-01

**Authors:** S. Charpentier Mora, C. Bastianoni, M. Tironi, F. Bizzi

**Affiliations:** University of Genoa, Department Of Educational Sciences (disfor), Genoa, Italy

**Keywords:** Covid-19, Emotion dysregulation, mentalizing, emerging adults

## Abstract

**Introduction:**

The COVID-19 pandemic represents an epidemiological and psychological crisis (APA, 2020). In this context, although emerging adults are less likely to get COVID-19, they might have suffered from the national lockdowns over the last year, as they are indeed involved in a crucial development period wherein interpersonal relationships undertake a fundamental function in their psychological well-being. To this end, mentalizing abilities and emotion dysregulation may play a crucial role as possible salutogenic or pathogenic factors on the onset of psychiatric symptoms during the three waves of the COVID-19 pandemic.

**Objectives:**

1) To examine the relationship between emotion dysregulation assessed at the end of the first wave of COVID-19, mentalizing assessed during the second wave, and psychiatric symptoms levels assessed during the third wave. 2) To examine the moderation role of mentalizing within the relation between emotion dysregulation and psychiatric symptoms.

**Methods:**

Participants were 83 non-clinical emerging adults (M_age_=22.18, SD=4.36; 57.8% females). Measures applied were Difficulties in Emotion Regulation Scale (DERS) to examine emotion dysregulation, Reflective Functioning Questionnaire to examine mentalizing (RFQ_uncertainty; RFQ_certainty) and Symptom Checklist-90-Revised (SCL-90) to examine psychiatric symptoms (Global Severity Index, GSI).

**Results:**

DERS_total score (r=.31, p=.03) and both RFQ_uncertainty (r=.41, p<.01) and RFQ_certainty (r=-.33, p=.02) are associated with GSI. Secondly, a significant moderation role by RFQ_u emerged within the relation between DERS_total score and GSI (∆R^2^=.067, β=.001, SE=.00, CI[.000, .002]).

**Conclusions:**

These results suggest that mentalizing and emotion dysregulation may play a pivotal role in the onset of psychiatric symptoms during the COVID-19 pandemic. Clinical implications are discussed.

**Disclosure:**

No significant relationships.

